# CHARACTERIZATION OF BASELINE SYMPTOMS AND FUNCTIONAL IMPAIRMENTS IN A LARGE COHORT OF OUTPATIENTS ATTENDING A LONG COVID REHABILITATION CLINIC IN THE UNITED KINGDOM

**DOI:** 10.2340/jrm-cc.v7.39984

**Published:** 2024-10-30

**Authors:** Matthew B. DOWNER, Emma TUCKER, Emily FRASER, Anton PICK

**Affiliations:** 1 Memorial University of Newfoundland, St. John’s NL, Canada; 2 University of Oxford, Oxford, United Kingdom; 3 Oxford Health NHS Foundation Trust, Oxford, United Kingdom; 4 Oxford University Hospitals NHS Foundation Trust, Oxford, United Kingdom

**Keywords:** post-acute COVID-19 syndrome, rehabilitation, retrospective studies

## Abstract

**Objective:**

In response to the high prevalence and morbidity associated with long COVID (LC), outpatient rehabilitation programmes were created across jurisdictions. We aimed to characterize baseline symptoms and impairments of patients attending outpatient LC rehabilitation.

**Design:**

This study was a retrospective quality-improvement analysis.

**Subjects/Patients:**

Patients attending outpatient LC rehabilitation at the Oxfordshire Post-Covid Service.

**Methods:**

Data included age/sex and 6 questionnaires performed at baseline: Functional Assessment of Chronic Illness Therapy-Fatigue (FACIT-F), Dyspnoea-12 (D12), Patient Health Questionnaire-9 (PHQ-9), Generalized Anxiety Disorder Assessment-7 (GAD-7), Visual Analogue Scale (VAS) of self-rated health, and the Work And Social Adjustment Scale (WSAS). All scores were dichotomized (indicating presence/absence of clinically significant pathology). Potential differences between age (</≥ 50 years) and sex were assessed using χ^2^ tests.

**Results:**

A total of 422 patients were included (mean/standard deviation [SD] age = 47.1/13.2;132/31.3% male). A total of 76% had significant fatigue (FACIT-F), 69% had breathlessness (D12), 55% had depression (PHQ-9), 34% had anxiety (GAD-7), 41% self-reported poor health (VAS), and 57% had work/social life dysfunction (WSAS). D12 scores differed between age groups (older > younger, χ^2^ = 3.19/*p* = 0.048), with no differences observed on other scales.

**Conclusion:**

In this preliminary study, a high proportion of LC outpatients had significant impairments across domains. The findings of this study reaffirm the need for high-quality, multidisciplinary LC rehabilitation, and may be used to help build a standardized set of outcome measures moving forward.

## LAY ABSTRACT

Long COVID (aka post-acute COVID-19 syndrome) occurs when people experience long-term, debilitating symptoms for several weeks to months after the initial infection from SARS-CoV-2. To try to help patients recover, health systems around the world have started multidisciplinary long COVID rehabilitation clinics. However, as these clinics are new, they have not yet been adequately studied.

In this study, we analysed over 400 patients who attended the Oxfordshire Post-Covid Service. At their first appointment, they were given 6 questionnaires that aimed to quantify symptoms in the following areas: fatigue, breathlessness, depression, anxiety, self-rated overall health, and work/social life dysfunction.

We found that long COVID patients attending this clinic had significant symptoms across all the domains assessed on the 6 questionnaires. Therefore, our work reinforces the need for long COVID rehabilitation programmes, and suggests these questionnaires could be implemented at long COVID rehabilitation clinics and research centres elsewhere.

As of December 2023, there have been over 770 million confirmed cases and approximately 7 million deaths due to COVID-19 (1). Long COVID (LC; also referred to as ‘post COVID-19 condition’) has emerged as a longer-term complication and can cause substantial functional impairment (2). Multiple definitions have been suggested for LC, with many describing patients with symptoms of COVID-19 that persist for at least 12 weeks after an initial infection with SARS-CoV-2 (2, 3). A wide range of LC symptoms have been reported, including fatigue, cognitive problems (difficulty concentrating, memory issues, etc.), breathlessness, generalized aches and pains, among others (2, 4). Furthermore, recent evidence suggests that LC represents a wide range of clinical phenotypes, with patients presenting with various symptoms, functional limitations, and rehabilitation requirements (3, 4).

Recent estimates from the *Office of National Statistics* suggest that 1.9 million people in the UK are living with LC (~2.9% of the population), with 79% reporting significant impairment in daily activities (5). A recent systematic review including 194 studies and 735,006 LC patients reported that 45% of patients who survived COVID-19 had at least 1 persisting symptom at a median follow-up of approximately 4 months (2).

In response to the high prevalence of LC and substantial functional impairments associated with the condition, rehabilitation for LC has become a research priority, with a wide array of multidisciplinary rehabilitation programmes having been created and implemented globally (6, 7). However, LC rehabilitation remains understudied, and research on best practices for LC rehabilitation is in its early stages (6). Additionally, most data on long COVID incidence and the prevalence of LC symptoms is based on administrative, primary care, or hospital-based datasets (2, 4), with fewer data on patients undergoing outpatient LC rehabilitation. Therefore, an improved understanding of patients referred to outpatient LC rehabilitation may be helpful to better characterize baseline impairments, which in turn can be used to inform future health system planning, improve national and international consensus on instruments to employ in LC rehabilitation, and help identify priority areas for future LC rehabilitation programmes.

The Oxfordshire Post Covid Service was among the first programmes set up for LC rehabilitation in the UK in 2020. In this preliminary report, we aim to outline the baseline characteristics of over 400 LC patients referred for outpatient rehabilitation in Oxfordshire.

## METHODS

### Patient ascertainment and clinic setup

Data was obtained from a quality improvement project based out of the *Oxfordshire Post Covid Service Assessment Clinic*. All patients were interviewed by clinic physicians between September 2020 and May 2023. Patients with persistent symptoms (> 12 weeks) were mostly referred by local general practitioners. The diagnosis of LC was made in accordance with recommendations by the *National Institute for Health and Care Excellence* (NICE) (8). NICE is the national body providing clinical guidelines in the UK (8). It has been publishing and regularly updating ‘rapid guidelines’ for the assessment and management of patients with the long-term effects of COVID-19 since December 2020. The guidance includes a standard definition and diagnostic criteria for the condition, summarized as ‘signs and symptoms that develop during or after an infection consistent with COVID 19, continue for more than 12 weeks and are not explained by an alternative diagnosis (8)’.

Data was collected only on patients who had a definite diagnosis of LC, and who were investigated for other causes of symptoms. Any patients that had other causes of symptoms, and thus were not diagnosed with LC, were excluded from the present analysis. With regard to baseline respiratory symptoms, patients with pre-existing respiratory disease were also excluded (apart from mild, well-controlled asthma).

Initial patient assessment included basic demographic information (age/sex) and a series of questionnaires to ascertain baseline symptoms across a range of domains. This included the following scales:

- *Functional Assessment of Chronic Illness Therapy (FACIT-F) Fatigue scale:* A 13-item questionnaire designed to measure an individual’s level of fatigue (9).
- *Dyspnoea-12 (D12):* A questionnaire designed to measure the severity of breathlessness in patients (10).
- *Patient Health Questionnaire-9 (PHQ-9):* A brief, self-administered questionnaire used to diagnose and monitor depression (11).
- *Generalized Anxiety Disorder Assessment-7 (GAD-7):* A measure of the level of anxiety and used to diagnose generalized anxiety disorder (12).
- *Visual Analogue Scale (VAS):* A scale on a 10cm line that asked participants to rate their current health on a scale of 0 (worst possible health) to 100 (best possible health) (13).
- *Work And Social Adjustment Scale (WSAS):* An instrument that assesses a range of functioning across multiple aspects of daily life, including work, home, social life, family, and personal commitments (14).

The outcome measures were selected by consensus by the clinicians working at the clinic. The rationale for the scales was that the clinicians believed all chosen scales were relevant for LC and enabled clinic staff to obtain a subjective measure of symptom burden in relation to fatigue and breathlessness (FACIT-F, D12), impact on mood (PHQ9) and anxiety (GAD7), as well as effect on daily living, participation, and functional activity (WSAS).

This project was approved by standard hospital governance processes as a service evaluation project and was therefore deemed not to require ethical approval. Data was handled in accordance with data protection protocols within the hospital and anonymized prior to all analyses.

### Statistical analyses

Continuous data were reported as mean (standard deviation [SD]), and categorical data as count (percentage-%). Potential differences between baseline demographic variables or questionnaire scores were assessed using *t*-tests, Mann-Whitney *U* tests, or χ^2^ tests, as appropriate.

To examine the proportion of patients with clinically significant symptoms on presentation, scores from each of the questionnaires were dichotomized to indicate the presence or absence of moderate to severe symptoms within a given domain. The following dichotomizations were used for the present study:

- *FACIT-F:* clinically significant fatigue: < 30; not clinically significant: ≥ 30 (9).
- *D12:* moderate to severe dyspnoea: > 3; none to mild dyspnoea: 0–3. These patients did not have a history of prior breathlessness or significant respiratory disease (10).
- *PHQ-9:* moderate-to-severe depression: ≥ 10; minimal to mild: < 10 (11).
- *GAD-7:* moderate-to-severe anxiety: ≥ 10; minimal to mild: < 10 (12).
- *VAS:* very poor overall self-rated health: 0–30; moderate to good: > 30 (13).
- *WSAS:* moderate-to-severe dysfunction ≥ 21; minimal to no dysfunction: < 21 (14).

Further, we aimed to assess potential differences in baseline symptomatology across domains between different sexes and age groups (</≥ 50 years) using the χ^2^ test. For the present analysis, we used complete case analysis and any patient with missing or incomplete data was excluded.

Significance was set at *p* < 0.05. Stata (V16; College Station, USA) was used for all analyses.

## RESULTS

A total of 607 patients attended the *Oxfordshire Post Covid Clinic* between January 2021 and May 2023. One hundred eighty-five patients were excluded from the present analyses, as they either did not answer all questions in the scales or wished to not complete the scales entirely. Four hundred twenty-two patients had complete data available and were included for analysis. The mean age was 47.1 years (SD 13.2), and 132 (31.3%) were male (Table I).

**Table I:** Baseline demographics and questionnaire responses in 422 long COVID patients at the OUH Long COVID Outpatient Rehabilitation Clinic (overall and stratified by age)

Variable/Instrument	Age ≤ 50	Age>50	Total	*p*
	*N* = 235	*N* = 187	*N* = 422	
*Age*	37.6 (8.3)	58.9 (7.2)	47.1 (13.2)	
*Male sex*	66 (28.1%)	66 (35.3%)	132 (31.3%)	0.11
*FACIT*	23 (18–29)	22 (15–28)	23 (17–29)	0.22
*D12*	7 (2–13)	9 (3–16)	8 (3–15)	**0.016**
*PHQ9*	10 (7–14)	11 (6–15)	10 (6–15)	0.70
*GAD7*	7 (3–12)	6 (3–11)	7 (3–12)	0.17
*VAS*	40 (8–80)	50 (9–80)	42.5 (8–80)	0.18
*WSAS*	22.5 (15–30)	23 (14–30)	23 (15–30)	0.98

FACIT: Functional Assessment of Chronic Illness Therapy; D12: Dyspnoea-12; PHQ-9: Patient Health Questionnaire-9; GAD-7: Generalized Anxiety Disorder Assessment-7; VAS: Visual Analogue Scale; WSAS: Work and Social Adjustment questionnaire.

Potential differences between age groups were assessed using *t*-tests, Mann-Whitney *U* tests, or χ^2^ test, as appropriate.

Data presented as mean (SD), median (IQR), or *n* (%). *P*-value represents difference between age groups. Significance was set at *p*<0.05.

**Fig. 1:**
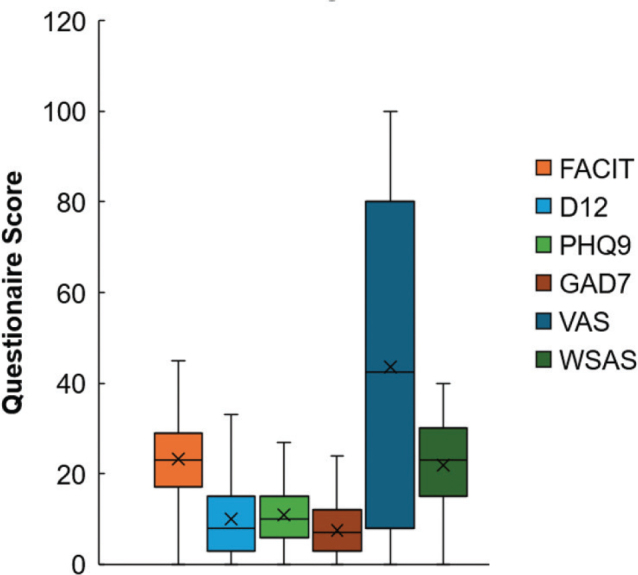
Distribution of scores across 6 baseline questionnaires in a cohort of 422 long COVID patients presenting to an outpatient long covid rehabilitation clinic. FACIT: Functional Assessment of Chronic Illness Therapy; D12: Dyspnoea-12; PHQ-9: Patient Health Questionnaire-9; GAD-7: Generalized Anxiety Disorder Assessment-7; VAS: Visual Analogue Scale; WSAS: Work and Social Adjustment questionnaire.

**Fig. 2:**
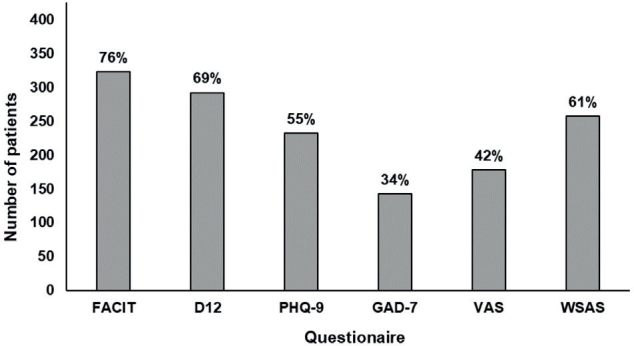
Number and proportion of long COVID patients with clinically significant impairments or moderate-to-severe pathology across 6 baseline questionnaires. FACIT: Functional Assessment of Chronic Illness Therapy; D12: Dyspnoea-12; PHQ-9: Patient Health Questionnaire-9; GAD-7: Generalized Anxiety Disorder Assessment-7; VAS: Visual Analogue Scale; WSAS: Work and Social Adjustment questionnaire.

Overall, the median scores on all the questionnaires used reflect a high degree of symptomatology and considerable functional impact across a wide range of domains (Table I, Fig. 1). With regard to our dichotomized analysis, 76% of patients had clinically significant fatigue (FACIT-F), 69% had breathlessness symptoms (D12), 55% had moderate-to-severe depression (PHQ-9), 34% had moderate-to-severe anxiety (GAD-7), 41% had very poor self-rated overall health (VAS), and 57% had moderate-to-severe dysfunction in work and social life (WSAS; Fig. 2).

**Fig. 3:**
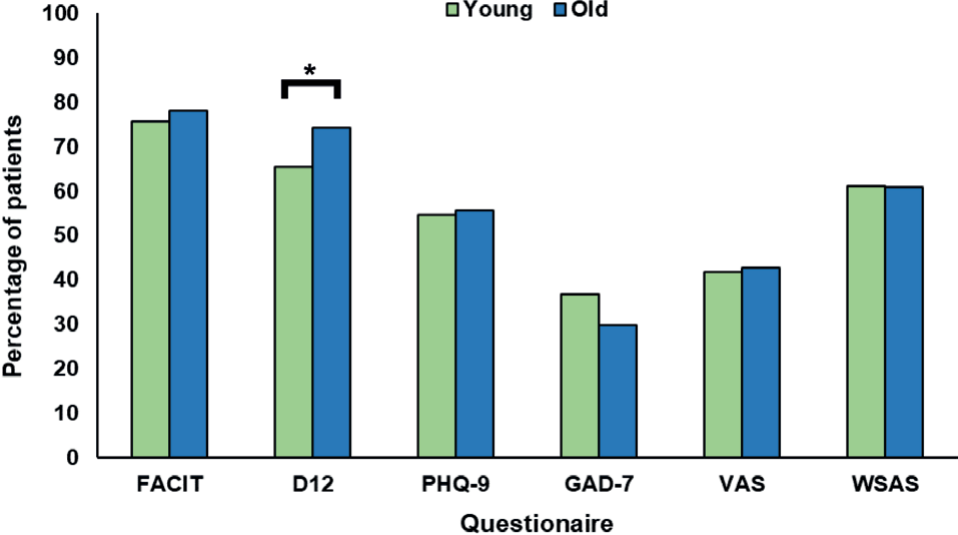
Proportion of long COVID patients with clinically significant impairments or moderate-to-severe pathology across 6 baseline questionnaires, stratified by age. *p < 0.05 between groups; using χ^2^ test. All other *p > 0.05.* FACIT: Functional Assessment of Chronic Illness Therapy; D12: Dyspnoea-12; PHQ-9: Patient Health Questionnaire-9; GAD-7: Generalized Anxiety Disorder Assessment-7; VAS: Visual Analogue Scale; WSAS: Work and Social Adjustment questionnaire. Young = less than 50 years of age; Old = 50 years of age and older.

**Fig. 4:**
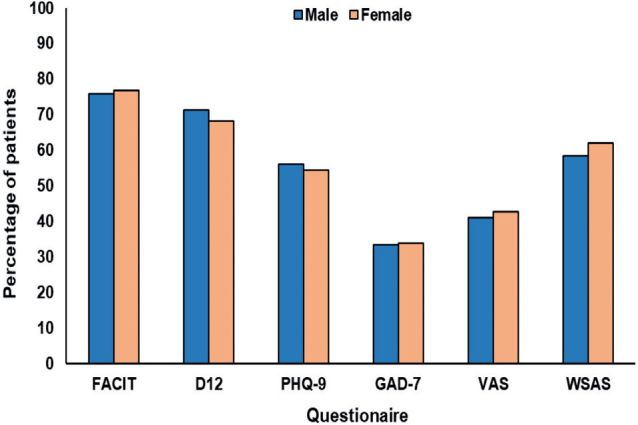
Proportion of long COVID patients with clinically significant impairments or moderate-to-severe pathology across 7 baseline questionnaires, stratified by sex. *p < 0.05 between groups; using χ^2^ test. All other *p > 0.05.* FACIT: Functional Assessment of Chronic Illness Therapy; D12: Dyspnoea-12; PHQ-9: Patient Health Questionnaire-9; GAD-7: Generalized Anxiety Disorder Assessment-7; VAS: Visual Analogue Scale; WSAS: Work and Social Adjustment questionnaire.

On stratified analysis, there were few differences in observed baseline scores between age groups (</≥ 50 years) and sex (male/female; Figs. 3 and 4). With regard to age, scores on the D12 were the only to differ across age groups, with older patients more likely to experience moderate to severe dyspnoea (χ^2^ = 3.19, *p =* 0.048; all others *p >* 0.05). For sex, there were no differences in scores across any of the scales used (all *p >* 0.05).

## DISCUSSION

In this study of over 400 patients who attended an outpatient LC clinic in the UK, we report a high proportion of significant impairments across a range of functional domains.

These findings align with previous work on LC (2, 4, 15–17). However, most studies on LC used large administrative, primary care, and/or hospital databases (2, 4), with relatively few data in the outpatient rehabilitation setting. This is one of the first reports characterizing baseline symptoms and impairments in patients referred to an outpatient LC rehabilitation programme. Three existing studies on patients attending outpatient LC rehabilitation were small (all *n* ≤110), did not do age/sex-stratified analyses, and did not include many of the questionnaires used in the present report (15–17).

There are some important implications of the current study.

First, our work underscores the importance and need for multidisciplinary rehabilitation for LC (6,7). In this outpatient setting, there was a very high prevalence of significant impairment in a range of domains, including fatigue, dyspnoea, anxiety, depression, poor self-reported health, and problems in work/social functioning. As such, this study reaffirms the need for high-quality, multidisciplinary outpatient rehabilitation for LC patients.

Second, this preliminary data can be used to contribute to efforts to create standardized LC rehabilitation programmes. To compare outcomes across centres and jurisdictions, a core set of outcome measures must be established to compare rates of symptoms, perform case-mix adjustment, and improve understanding of outcomes/trajectories in patients living with LC. For instance, a recent international consensus study for LC outcomes (*n* = 594 participants; multidisciplinary LC experts and patients with lived experience) reached consensus on outcome measures for survival, recovery, and respiratory outcomes, but did not reach consensus on the other 9 outcomes considered, including fatigue, work/occupational changes, mental health, and others (18). In the present study, we present baseline data on 6 scales/questionnaires on a large sample of LC patients in the outpatient setting. Based on the high prevalence of significant pathology and impairment on presentation captured by these scales, future guidelines aimed at setting up LC outpatient services, as well as future trials on LC, could consider implementing some of these measures.

There are multiple limitations to the present work that should be noted. First, we had limited data on demographics beyond age and sex, and additional information on hospitalization status, deprivation, employment, education status, as well as comorbidities, would have added additional granularity to our analyses. Second, this was not a population-based study, and only included patients with complete data, which may have biased the present findings. Third, we did not have follow-up data available for this study. However, some strengths include the large sample size, the multiple scales employed, and the stratification of analyses by age and sex.

In conclusion, in this preliminary study, we report that a high proportion of patients attending an outpatient LC clinic have significant impairments across a range of functional domains. However, there were little differences between age groups or sexes. This study reaffirms the need for high-quality, multidisciplinary rehabilitation for LC patients in outpatient settings, and may help future identification of a standardized set of scales used across jurisdictions for outpatient LC rehabilitation.
